# Two new species of the leafhopper subgenus *Empoasca* (*Empoasca*) Walsh (Hemiptera, Cicadellidae, Typhlocybinae, Empoascini) from China

**DOI:** 10.3897/zookeys.437.7563

**Published:** 2014-08-28

**Authors:** Xiaofei Yu, Maofa Yang

**Affiliations:** 1 Institute of Entomology, Guizhou University, Guiyang Guizhou, 550025, P. R. China; 2 Guizhou Provincial Key Laboratory for Agricultural Pest Management of the Mountainous Region, Guiyang Guizhou, 550025, P. R. China.

**Keywords:** Subgenera, Key

## Abstract

Two new species of the leafhopper subgenus *Empoasca* (*Empoasca*) namely, *E.* (*E.*) *dorsodenticulata* Yu & Yang, **sp. n.** and *E.* (*E.*) *spiculata* Yu & Yang, **sp. n.** from China are described and illustrated and a key provided for Chinese subgenera of *Empoasca*.

## Introduction

The leafhopper genus *Empoasca* Walsh, 1862 includes 11 subgenera (Oman et al. 1990) of which five occur in China: *Empoasca (Empoasca)* Walsh, 1862, *Empoasca (Distantasca)* Dworakowska, 1972, *Empoasca (Matsumurasca)* Anufriev, 1973, *Empoasca (Livasca)* Dworakowska & Viraktamath, 1978 and *Empoasca (Okubasca)* Dworakowska, 1982. The subgenus *Empoasca (Empoasca)* is very species rich with over 400 known species worldwide of which 53 species are known from China treated by Dworakowska (1972, 1982), Kuoh (1966), Chou and Ma (1981), Cai and Shen (1999), Matsumura (1931), Zhang and Xiao (2000), Zhang, Liu and Qin (2008), Liu, Qin, Fletcher and Zhang (2011a, 2011b) and Yu, Yang and Meng (2012). In the current paper we describe two new species in the subgenus from China and provide a key to the subgenera of *Empoasca* from China.

## Material and methods

The methods and terminology follow Zhang (1990) except for the nomenclature of wing, for which we follow Dworakowska (1993). Male specimens were dissected under a MOTIC B1 SMS-168 SERIES microscope. Figures were made using an OLYMPUS CX41 and enhanced using Adobe Illustrator CS4. Pictures were taken with VHX-1000C and dealt with by Adobe Illustrator CS4. The body length is measured from the apex of the head to the apex of the forewing, the specimens examined are deposited in Institute of Entomology, Guizhou University, Guizhou Guiyang, China (GUGC) and The Natural History Museum, England.

## Results

### Empoasca (Empoasca)

Taxon classificationAnimaliaHemipteraCicadellidae

Walsh, 1862

Empoasca (Empoasca) Walsh, 1862: 149

#### Type species.

*Empoasca viridescens* Walsh (a junior synonym of *Tettigonia fabae* Harris, 1841).

#### Diagnosis.

**Figures 1–8.**
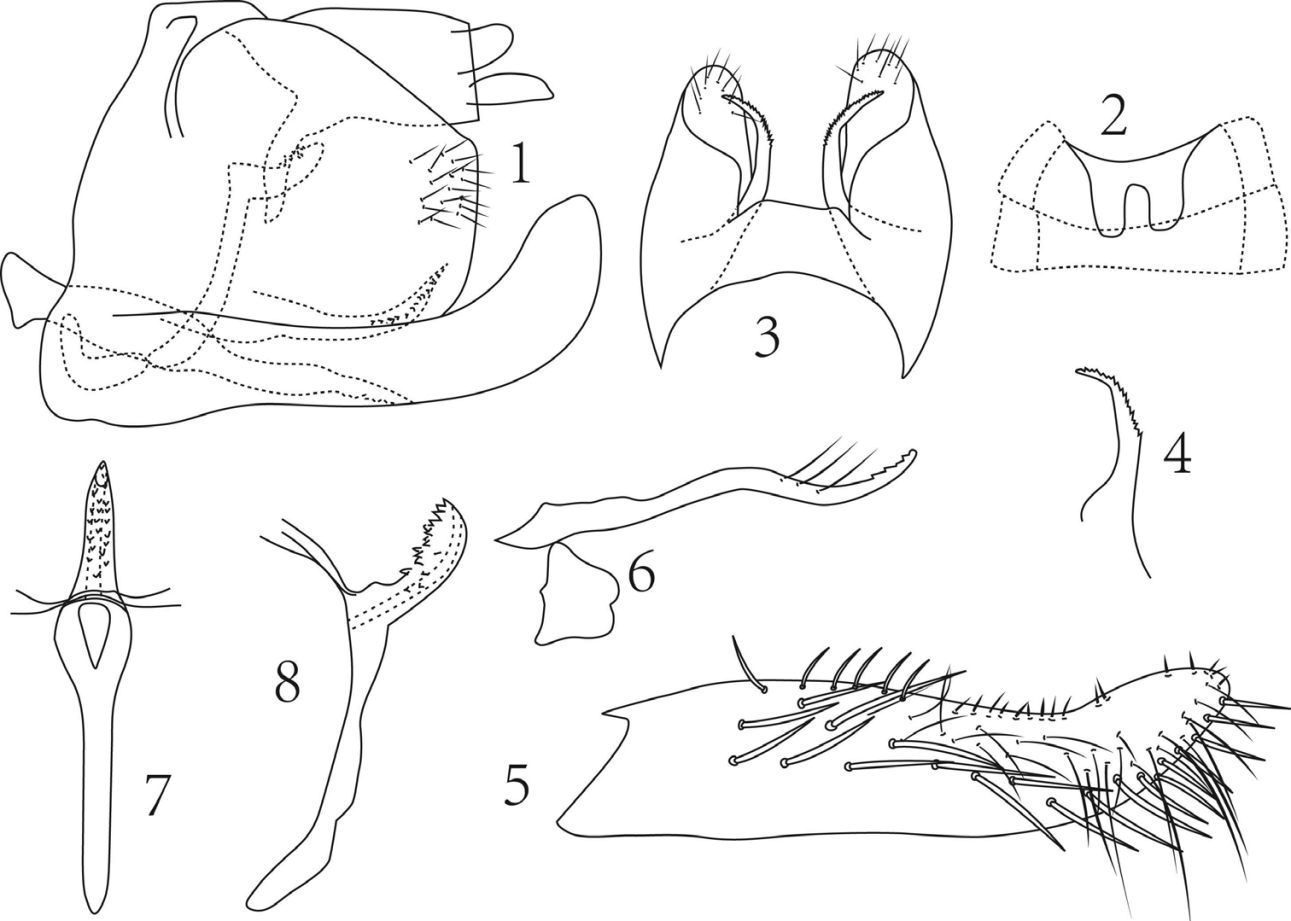
*Empoasca (Empoasca) dorsodenticulata* Yu & Yang, sp. n. **1** male genital capsule, lateral view **2** male abdominal apodemes **3** male pygofer, dorsal view **4** ventral pygofer appendage, dorsal view **5** subgenital fig, ventral view **6** paramere and connective **7** aedeagus, dorsal view **8** aedeagus, lateral view.

**Figures 9–17.**
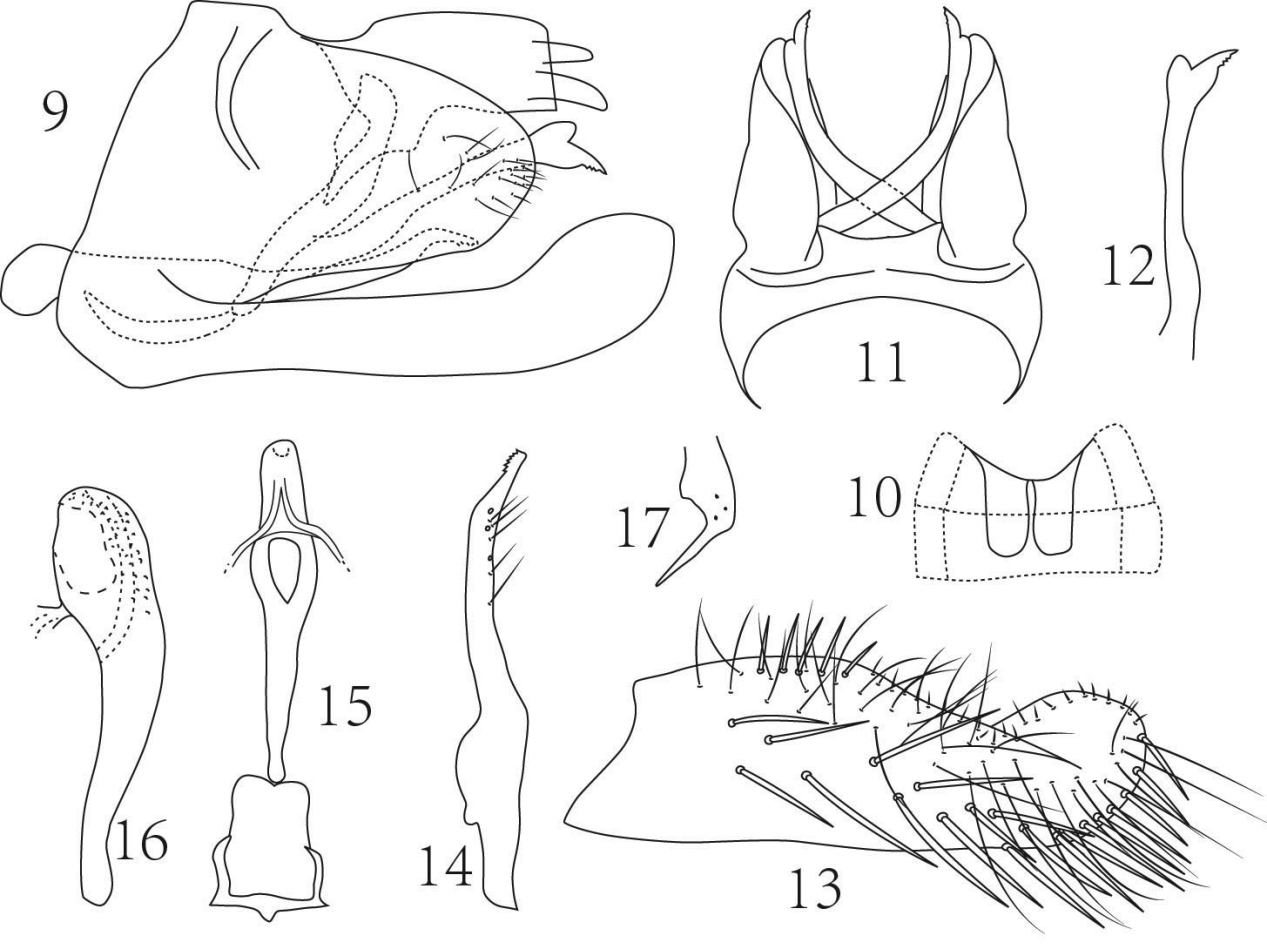
*Empoasca (Empoasca) spiculata* Yu & Yang, sp. n. **9** male genital capsule, lateral view **10** male abdominal apodemes **11** male pygofer, dorsal view **12** ventral pygofer appendage, dorsal view **13** subgenital fig, ventral view **14** paramere **15** aedeagus and connective, dorsal view **16** aedeagus, lateral view **17** anal tube process.

**Figures 18–23.**
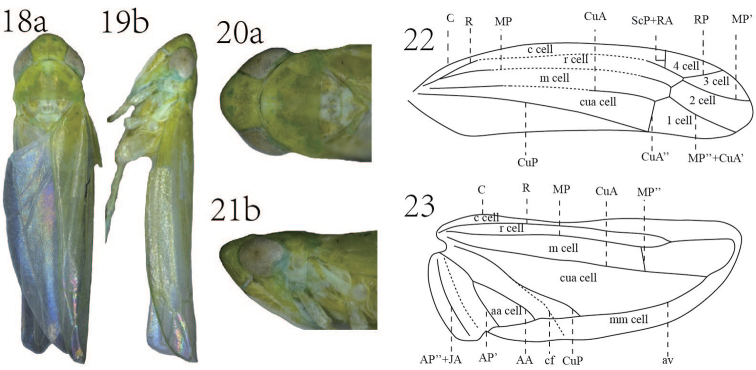
*Empoasca (Empoasca) dorsodenticulata* Yu & Yang, sp. n. **18–19** whole body **20–21** head and thorax **22** forewing **23** hindwing **a** dorsal view **b** lateral view.

**Figures 24–29.**
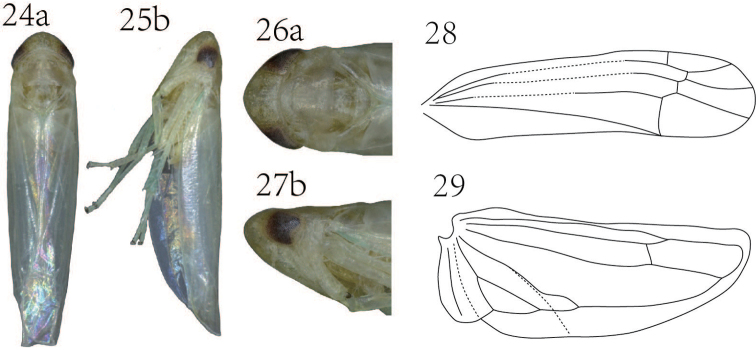
*Empoasca (Empoasca) spiculata* Yu & Yang, sp. n., **24–25** whole body **26–27** head and thorax **28** forewing **29** hindwing **a** dorsal view **b** lateral view.

Body color green to yellowish, with variable symmetrical patches on head and thorax; coronal suture not reaching midlength of crown (Figs 18a, 20a, 24a, 26a); forewing with RP and MP’ stalked (Fig. 22) or separated (Fig. 28), hindwing with CuA unbranched (Fig. 23, 29); male pygofer, with fine sparse setae distally, macrosetae absent, ventral pygofer appendage present (in some species processes crossed in dorsal view), free from pygofer lobe, at least for halflength of pygofer (Figs 1, 9); subgenital fig broad basally, basal series of setae on outer margin and an oblique series of macrosetae from base to apex bi-seriate basally (Figs 5, 13); paramere elongate, curved, with apical teeth (Figs 6, 14); aedeagus with preatrium present, shaft without processes or occasionally with pair of processes, gonopore apical on ventral surface, dorsal apodeme poorly developed or absent (Figs 7, 8, 15, 16); anal tube processes distinct.

#### Distribution.

Worldwide.

### Empoasca (Empoasca) dorsodenticulata

Taxon classificationAnimaliaHemipteraCicadellidae

Yu & Yang sp. n.

http://zoobank.org/D485282D-C23B-4CC4-B628-22179ACC251A

Figs 1–8 , 18 –23

#### Type material.

Holotype: male, Kuankuoshui, Guizhou Province, 14 August 2010, coll. Xiaofei Yu. Paratypes: 1 male, Shiwandashan, Guangxi Province, 4 May 2011, coll. Rong Huang; 1 male, Liupanshui, Guizhou Province, 2 June 2012, coll. Maofa Yang, Zhijiang Bai and Xiaofei Yu.

Length. Male: 4.0–4.1mm.

Green to yellowish (Fig. 18a). Crown with a green patch each side of coronal suture (Figs 18a, 20a). Face in some species with an off-white longitudinal stripe on anteclypeus (Figs 19b, 21b). Scutellum with a whitish stripe medially (Figs 18a, 20a). Forewing with RP and MP’ stalked at base (Fig. 22).

Male ventral abdominal apodemes reaching segment 4 (Fig. 2). Male pygofer lobe with dorsal margin oblique and apical margin straight in lateral view, with fine setae adjacent apical margin; ventral pygofer appendage shorter than pygofer, tapering caudad, subapically denticulate; dorsal bridge about 1/3 length of pygofer (Figs 1, 3, 4). Subgenital figs elongate, with 20 macrosetae arranged obliquely in two basal rows centrally and a single distal row on lateral margin, ca. 25 elongate fine setae and medial margin with 6 basal group macrosetae followed by ca. 16 spine-like setae (Figs 1, 5). Paramere as in Fig. 6. Aedeagus with elongate preatrium; shaft slightly expanded near apex in lateral view, tapered from base to apex in ventral view, apical 1/3 with numerous irregular denticles dorsally (Figs 1, 7, 8). Anal tube process slightly sinuate (Fig. 1). Connective lamellate (Fig. 6).

#### Etymology.

The new species name alludes to the dorsal denticles on the aedeagal shaft.

#### Remarks.

The new species is similar to *Empoasca (Empoasca) borowikae* Dworakowska, 1976 but differs in having the male ventral abdominal apodemes reaching segment 4, ventral pygofer appendage denticulate near apex and apical 1/3 of aedeagus with dorsal denticles. The new species is also similar to *Empoasca (Empoasca) gutianensis* Liu, 2011 but differs in the aedeagus without denticles ventrally and anal tube process sinuate.

### Empoasca (Empoasca) spiculata

Taxon classificationAnimaliaHemipteraCicadellidae

Yu & Yang sp. n.

http://zoobank.org/4CDD261B-E282-4D5F-9F8F-26BA4CA306B2

Figs 9 –17 , 24 –29

#### Type material.

Holotype. male, Luya mountain, Shanxi Province, 19 August 2011, coll. Hu Li; Paratypes: 5 males, Lvliang mountain, Shanxi Province, 22 August 2011, coll. Hu Li, Zhihua Fan and Xiaofei Yu (1 male, BMNH).

Length. Male 3.9–4.1 mm.

Yellowish (Fig. 24a). A yellow stripe along coronal suture (Figs 24a, 26a). Scutellum with a central whitish streak (Figs 24a, 26a). Forewing with RP and MP’ stalked at base or separated (Fig. 28).

Male ventral abdominal apodemes reaching segment 4 (Fig. 10). Male pygofer lobe tapered to rounded apex with ca. 15 setae, ventral pygofer appendage extended far beyond pygofer, procceses crossed in dorsal view, apex expanded and forked, lower branch serrate; dorsal bridge about 1/4 length of pygofer (Figs 9, 11, 12). Subgenital figs relatively broad with 20 macrosetae arranged obliquely in two basal rows centrally and a single distal row on lateral margin, and ca. 37 elongate fine setae from base to apex and medial margin with 5 basal group macrosetae followed by ca. 22 spine-like setae (Figs 9, 13). Paramere as Fig. 14. Aedeagus club-shaped in lateral view; shaft dorsally laterally compressed and less sclerotized, ventrally spiculate, with apex broadly rounded in ventral view; (Figs 9, 15, 16). Anal tube process falcate, apex spine-like (Figs 9, 17). Connective lamellate (Fig. 15).

#### Etymology.

The new species name alludes to the ventral spicules on the aedeagal shaft.

#### Remarks.

The new species differs from other members of the subgenus in having the ventral pygofer appendage forked, aedeagal shaft laterally compressed dorsally and ventrally spiculate.

### Key of the subgenera of *Empoasca* known from China (males only)

**Table:** 

1	Subgenital figs with very long fine setae distally (Fig. 30)	*Empoasca (Distantasca)*
–	Subgenital figs with macrosetae distally (Figs 31, 32, 33, 34)	2
2	Pygofer lobe with rigid microsetae at ventral margin and ventral pygofer appendage short; subgenital figs elongate and tapered to narrow apex, with all outer marginal setae short (Fig. 31)	*Empoasca (Okubasca)*
–	Pygofer lobe without rigid microsetae at ventral margin and ventral pygofer appendage long (Fig. 1); subgenital figs moderately long and tapered to broadly rounded apex, with outer basal group of long macrosetae and more distal short setae (Figs 32, 33, 34)	3
3	Subgenital figs very short and broad (Fig. 32)	*Empoasca (Livasca)*
–	Subgenital figs elongate	4
4	Subgenital figs distinctly broader basally than distally (Fig. 33)	*Empoasca (Matsumurasca)*
–	Subgenital figs slightly broader basally than distally (Fig. 34)	*Empoasca (Empoasca)*

**Figures 30–34.**
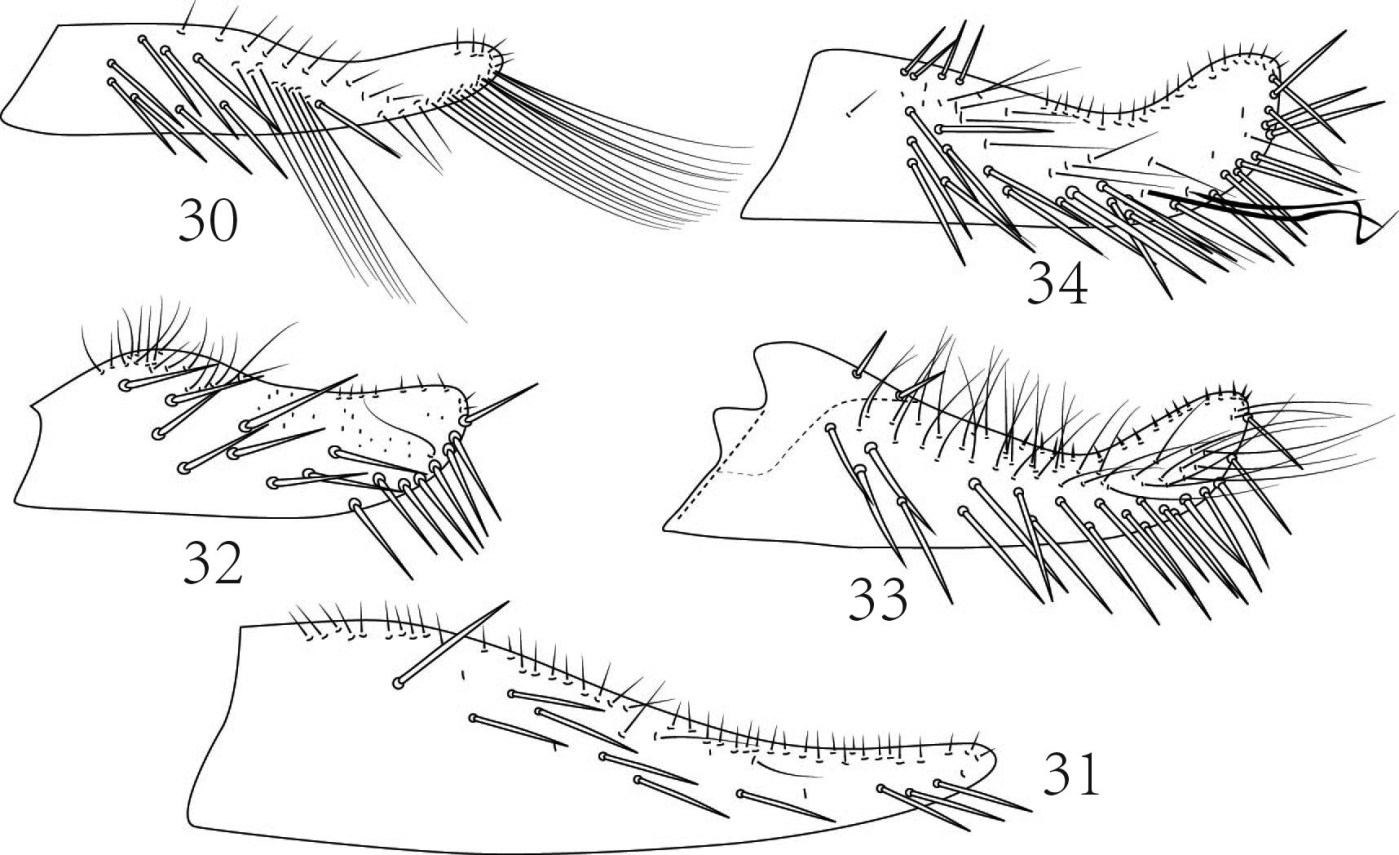
*Empoasca* subgenera from China, subgenital figs (type species except 33). **30** *Empoasca (Distantasca) terminalis* (following Distant 1918) **31** *Empoasca (Okubasca) okubella* (following Matsumura 1931) **32** *Empoasca (Livasca) malliki* (from Dworakowska and Viraktamath 1978) **33** *Empoasca (Matsumurasca) clypealata* (from Qin and Zhang 2008) **34** *Empoasca fabae* (from Harris 1841).

## Supplementary Material

XML Treatment for Empoasca (Empoasca)

XML Treatment for Empoasca (Empoasca) dorsodenticulata

XML Treatment for Empoasca (Empoasca) spiculata
